# Metal Fume Fever: An Underdiagnosis

**DOI:** 10.7759/cureus.34928

**Published:** 2023-02-13

**Authors:** Adriana Girao, José Abreu Fernandes, Leandro Valente, Cristiane Macedo, Rui Pina

**Affiliations:** 1 Internal Medicine, Centro Hospitalar e Universitário de Coimbra, Coimbra, PRT

**Keywords:** metal oxides, copper, fever, metal fume fever, metal fume

## Abstract

Metal fume fever (MFF) is an auto-limited acute febrile respiratory syndrome that may mimic an acute viral respiratory disease after exposure to the fumes of metal oxides. Due to the similar presentation of an influenza-like illness, it remains an underdiagnosed disease. It is typically a benign and self-limited entity that resolves over 12-48 hours following cessation of exposure, but symptoms may reoccur with repeated exposure. Supportive and symptomatic care is recommended.

## Introduction

Metal fume fever (MFF) is an acute febrile syndrome that presents as an influenza-like illness after the inhalation of metal fumes [1]. The primary cause is the inhalation of freshly formed zinc oxide fumes (most common) or other metal fumes such as copper, magnesium, tin, or cadmium [2]. MFF typically presents with non-specific complaints, including influenza-like symptoms, such as fever, shaking chills, arthralgias, myalgias, and headache, a metallic aftertaste, and abdominal discomfort. This usually occurs three to 10 hours after exposure to a variety of metal oxides [3,4]. Since MFF presents with clinical features that are alike to those caused by respiratory viruses, it is frequently misdiagnosed if an occupational history is not taken [3]. Symptoms usually resolve 12 to 48 hours after cessation of exposure [3,5].

## Case presentation

A 29-year-old male patient, with no previous medical history, presented to the emergency department (ED) after exposure to copper hydroxide by accidentally bursting powdered fungicide. At the time of the accident, he had no protective gear and admitted having unintentionally inhaled powder and not being able to specify the quantity. He presented in the ED four hours after exposure, with a temperature of 38.4ºC, chills, myalgias, watery diarrhea, and headache. Supportive and symptomatic care was initiated with fluid therapy and antipyretic drugs (paracetamol 1000 milligrams). The national poison center (CIAV - Centro de Informação Antivenenos) was contacted, and our team was advised to maintain supportive care and clinical and analytical surveillance given the risk of upper respiratory tract irritation and maintenance of a febrile state. It was suggested to do a serial measurement of serum copper concentration and methemoglobin and maintain supportive care. Serum copper concentration was on the upper normal level (1.39 mg/mL) and methemoglobin was within normal values (0.5%). All other bloodwork, examinations, and investigations were unremarkable. The patient remained under observation in the ED for 24 hours and during that time, he maintained hyperthermia (>38ºC). The administration of cold intravenous fluids and peripheral cooling was needed to sustain normothermia. Due to the necessity of clinical monitoring, he was admitted to an internal medicine ward, where he remained for four days. He progressively recovered, with no more myalgias and diarrhea and the need for antipyretic drug administration in the last 24 hours before discharge. A diagnosis of MFF after accidental exposure to copper hydroxide was made after consulting the existing literature.

## Discussion

The MFF pathogenesis relies on a non-specific, nonallergic activation of macrophages or pulmonary epithelial cells with a local and systemic release of pyrogenic and chemotactic mediators [4]. The mechanism of MFF is not completely understood, but it is thought to be associated with systemic neutrophilia and cytokine activation, usually with an increase in interleukin-6 and interleukin-8 [1]. Typically, the symptoms begin three to 10 hours after exposure and follow a benign and self-limited course. Severe disease is rare, but it has been reported, especially in patients with previous respiratory diseases [3,5]. Acute respiratory distress syndrome (ARDS) and the need for mechanical ventilation are rare. Imaging tests (chest radiography) are usually normal in cases of MFF; however, mild vascular congestion could be present, and in severe cases, diffuse patchy infiltrates and progression to ARDS can occur [5]. Usually, there is no need to perform laboratory studies in order to achieve a diagnosis but bloodwork may demonstrate leukocytosis and an increased erythrocyte sedimentation rate [5]. The evidence of metal fume exposure, clinical presentation, and the resolution of symptoms after cessation of exposure are essential hallmarks of diagnosis [6]. Evidence of possible exposure is critical [6]. Supportive and directed care for symptom relief is the primary treatment for MFF [5]. Anti-inflammatory drugs, such as non-steroidal drugs and antipyretics, are recommended as well as rest and oral hydration [5]. More severe cases may need hospitalization for intravenous hydration and cooling. Since MFF is a typically benign and self-limited disease, most of the symptoms resolve 12 to 48 hours after exposure [5].

## Conclusions

In hyperacute exposures rather than chronic, it is expected to find normal metal oxide levels. In this case, the patient presented a copper level in the upper limit of normal. Hyperacute exposures causing MFF are rare and despite being documented in the literature several years ago, MFF remains underdiagnosed given its similar presentation to an influenza-like illness. A proper medical history with an adequate occupational history is essential for diagnosis. Early recognition of symptoms and possible exposure can ensure a more directed management approach and exclude other serious inhalation illnesses.
